# The implementation in Europe (EU) of the low risk drinking guidelines: results from the RARHA survey

**DOI:** 10.1186/1940-0640-10-S2-P17

**Published:** 2015-09-24

**Authors:** Emanuele Scafato, Ghirini Silvia, Galluzzo Lucia, Gandin Claudia

**Affiliations:** 1 Istituto Superiore di Sanità, Italy

## Background

RARHA (REDUCING ALCOHOL RELATED HARM) is a Joint Action (JA) (2014-2016) that is funded under the EU Health Programme and by EU Member States to address some commonly identified priorities to reduce levels of alcohol related harm in the EU. The RARHA work package 5 contributes towards increased understanding among public health policy makers of the scientific basis and practical implications of the use of low risk drinking guidelines as a public health measure. The objectives are to evaluate the presence (or not) of low risk drinking guidelines (GL) o recommendations (R) in Europe on the basis of the existing and available EU documents (Drug and Alcohol Review; WHO additional survey 2012; WHO Status report on alcohol and Health in 35 EU countries; OECD Collection on national drinking guidelines) (1-4) and by additional information based on ad hoc survey across European Union Member States.

## Material and methods

A country specific questionnaire has been developed by the ONA. The form has been submitted by email to the country representatives of the Committee on National Alcohol Policy and Action (CNAPA) to check the validity of the information provided by the Country questionnaire and to provide the most updated and reliable information for their Country.

## Results

Twenty-eight countries have a definition of Standard Drink (SD) expressed as grams of pure alcohol but there isn't a consensus on how much alcohol is contained in one standard drink. Twenty-two countries have GL or R on low drinking guidelines for daily or weekly consumption and twenty countries have GL or R on binge drinking. Six countries have GL or R specific for the elderly and 15 countries for the youth. The majority of Member States have GL or R for alcohol consumption during pregnancy. In Europe the general drink driving limit is 0.5mg, but there are differences from country to country and in some countries there are specific limitations for young/novice drivers or professional/commercial drivers.

## Conclusions

The collected information represent the scientific basis and practical implications of the use of drinking guidelines as a public health measure. It serves to clarify the science underpinnings and practical/policy implications concerning low risk drinking guidelines, and work towards consensus on good practice principles in the use of drinking guidelines as a public health measure to help reduce HHAC. This activity, finalized to provide a more aligned messages to the general population, subgroups and health professionals, is ongoing (Rarha Delphi survey).
